# Recurrent Mixed Autoimmune Hemolytic Anemia With Evans Syndrome and High-Risk Relapse Features: A Case Report

**DOI:** 10.7759/cureus.108861

**Published:** 2026-05-14

**Authors:** Khiet T Nguyen, Kaung Htet Hla Win, Duong Huynh, Hitendra Rambhia, Kalpana Panigrahi, Madhumati R Kalavar, Edouard Guillaume

**Affiliations:** 1 Medicine, One Brooklyn Health/Interfaith Medical Center, Brooklyn, USA; 2 Internal Medicine, One Brooklyn Health/Interfaith Medical Center, Brooklyn, USA; 3 Internal Medicine, Wyckoff Heights Medical Center, Brooklyn, USA; 4 Hematology and Medical Oncology, One Brooklyn Health/Interfaith Medical Center, Brooklyn, USA; 5 Hemato-oncology, One Brooklyn Health/Brookdale University Hospital and Medical Center, Brooklyn, USA

**Keywords:** evans’ syndrome, immune check-point inhibitor, mixed autoimmune hemolytic anemia, recurrent aiha, retifanlimab

## Abstract

Autoimmune hemolytic anemia (AIHA) is a rare and heterogeneous disorder characterized by immune-mediated red blood cell destruction, often posing diagnostic and therapeutic challenges, particularly in patients with multiple comorbid conditions. We report a complex case of recurrent mixed AIHA occurring in the setting of Evans syndrome, human immunodeficiency virus (HIV) infection, metastatic malignancy, and recent exposure to an immune checkpoint inhibitor. The patient presented with profound anemia and laboratory evidence of hemolysis, with a positive direct antiglobulin test demonstrating both IgG and complement (C3) involvement, consistent with mixed AIHA. The clinical course was marked by multiple relapses despite initial treatment with corticosteroids, intravenous immunoglobulin, and transfusion support, ultimately requiring escalation to rituximab for sustained hematologic stabilization. Notably, the disease course demonstrated discordant recovery patterns between hemoglobin and platelet counts, reflecting complex immune dysregulation. This case highlights the challenges in managing mixed AIHA with overlapping risk factors and underscores the potential role of immune checkpoint inhibitors and chronic immune activation in precipitating or exacerbating autoimmune cytopenias. It also emphasizes the importance of early recognition of high-risk features, including severe anemia and concomitant thrombocytopenia, which may warrant prompt escalation to second-line therapy. Further investigation is needed to better define optimal treatment strategies and the underlying mechanisms driving relapse in this population.

## Introduction

Autoimmune hemolytic anemia (AIHA) is a rare form of anemia caused by immune-mediated destruction of red blood cells through autoantibodies of the IgG, IgM, or IgA subclasses, with or without complement activation [1]. Based on the thermal properties and immunologic characteristics of these antibodies, AIHA is classified into warm AIHA, cold agglutinin disease, mixed AIHA, and atypical variants [2,3].

Unlike anemia due to blood loss or nutritional deficiency, AIHA requires a structured diagnostic approach that incorporates laboratory confirmation of hemolysis and interpretation of the direct antiglobulin test to establish the immune etiology and subtype (Figure 3). Although AIHA may be primary, it is frequently secondary to infections, malignancy, medications, or autoimmune disorders. Identification of a secondary trigger may help guide management and may influence disease severity, relapse risk, and therapeutic response [1-3].

Here, we present a complex case of severe mixed AIHA with probable multifactorial secondary contributors, including HIV infection, active malignancy, and recent exposure to the immune checkpoint PD-1 inhibitor retifanlimab. The initial presentation was notable for profound anemia (hemoglobin <6 g/dL) and concomitant thrombocytopenia consistent with Evans syndrome, both established predictors of poor prognosis [4]. The disease course was further complicated by infection, kidney failure, and refractoriness to first-line therapy, necessitating multiple treatment modalities.

## Case presentation

The patient is a 66-year-old male with a history of HIV infection, chronic kidney disease (CKD) stage IV, hypertension, and anal intraepithelial neoplasia grade 3 (AIN3) with invasive foci, diagnosed in 2011. He was lost to follow-up for several years until August 2024, when he re-presented with an enlarging, friable perianal mass with intermittent bleeding. Biopsy confirmed invasive, moderately differentiated, p16-positive anal squamous cell carcinoma. His HIV was controlled on an outpatient basis with Biktarvy, and his CKD had a baseline serum creatinine of 3 to 4 mg/dL.

He underwent definitive chemoradiation from November to December 2024. Follow-up imaging then demonstrated partial tumor response but progressive left inguinal lymphadenopathy, with biopsy confirming recurrent metastatic squamous cell carcinoma. Systemic therapy with carboplatin-paclitaxel plus retifanlimab (PD-1 inhibitor) was initiated on August 19, 2025.

The patient presented to the emergency department on September 15, 2025, after several weeks of generalized weakness and persistent non-bloody diarrhea. On arrival, he was found to have severe anemia, with a hemoglobin level of 4.7 g/dL. Initial evaluation showed no overt acute bleeding. Laboratory studies (Table 1) revealed hemolysis, with low haptoglobin, elevated lactate dehydrogenase (LDH), indirect hyperbilirubinemia, and a strongly positive direct antiglobulin test (DAT) (IgG 3+), consistent with immune-mediated hemolysis. 

**Table 1:** Laboratory results of the day one on the first admission. CBC, complete blood count; WBC, white blood cell count; CD4, cluster of differentiation 4; CMP, comprehensive metabolic panel; CO₂, carbon dioxide; BUN, blood urea nitrogen; ALT, alanine aminotransferase; AST, aspartate aminotransferase; INR, international normalized ratio; HIV, human immunodeficiency virus; LDH, lactate dehydrogenase; CRP, C-reactive protein; aPTT, activated partial thromboplastin time; FOBT, fecal occult blood test; HBV, hepatitis B virus; HCV, hepatitis C virus; DAT, direct antiglobulin test; IgG, immunoglobulin G; C3, complement component 3.

CBC	Result	Reference Range
WBC	4.7	4.0–11.0 k/µL
Hemoglobin	4.9	13.5–17.5 g/dL
Hematocrit	14.4%	41–53%
Platelets	113	150–400 k/µL
CD4 count	221	500–1500 /µL
% CD4	24.6%	30–60%
CMP		
Sodium	141	135–145 mmol/L
Potassium	4.5	3.5–5.1
Chloride	110	98–107
CO₂ (bicarbonate)	21.2	22–29
Anion gap	10	8–16
Osmolality (calculated)	299.33	275–295
BUN	30	7–20 mg/dL
Creatinine	3.12	0.6–1.3 mg/dL
BUN/creatinine ratio	9.62	10–20
Glucose	119	70–99 mg/dL
Calcium	9.4	8.5–10.5 mg/dL
Corrected calcium	9.4	8.5–10.5 mg/dL
Total protein	7.2	6.0–8.3 g/dL
Albumin	4.0	3.4–5.4 g/dL
Globulin	3.2	2.0–3.5 g/dL
ALT	9	7–56 U/L
AST	26	10–40 U/L
Alkaline phosphatase	89	44–147 U/L
Total bilirubin	2.7	0.2–1.2 mg/dL
INR	1.1	0.9–1.1
Vitamin B12	658	200–900 pg/mL
Folate	9.6	>3 ng/mL
HIV viral load	30	Undetectable (<20–40)
Haptoglobin	<30	30–200 mg/dL
LDH	906	140–280 U/L
Iron	215	60–170 µg/dL
Iron saturation	93%	20–50%
Ferritin	2117	30–400 ng/mL
CRP	8.5 mg/dL	<0.5 mg/dL
Cold antibody	Positive	Negative
Non-specific antibody	Positive	Negative
aPTT	29.4 sec	25–35 sec
FOBT	Negative	
HBV	Negative	
HCV	Negative	
DAT IgG	Positive, 3+	Negative
DAT C3	Positive, 1+	Negative

Peripheral smear showed mild thrombocytopenia without clumping, hypochromic anemia with nonspecific anisopoikilocytosis (<1% schistocytes), and RBC left shift, suggesting a combination of marrow stress and hemolysis.

The patient was diagnosed with severe AIHA. He received pulse dexamethasone 40 mg daily for four days, intravenous immunoglobulin (IVIG) 1 g/kg for two days, and a total of seven units of washed, packed RBCs. Hemoglobin gradually improved, and he was discharged after eight days on a steroid taper with oncology follow-up.

**Figure 1:**
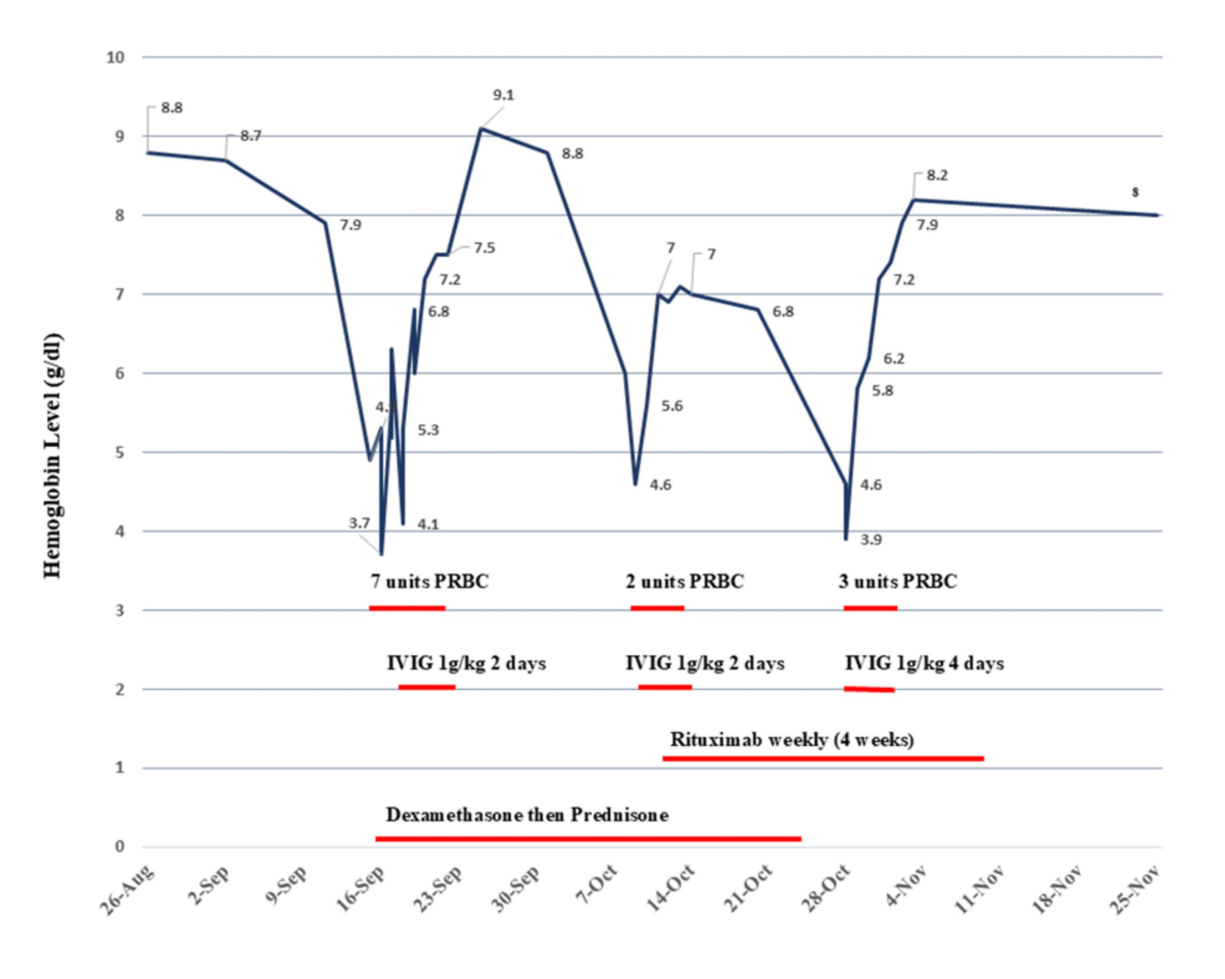
Hemoglobin trends and treatment timeline during three hospitalizations for recurrent mixed autoimmune hemolytic anemia.

Over the following months, he experienced two additional episodes of hemolysis requiring readmission, IVIG, corticosteroids, and initiation of rituximab. During the third episode, prolonged IVIG and a full four-week course of rituximab were required to achieve sustained stabilization of hemoglobin (Figure 1).

**Figure 2:**
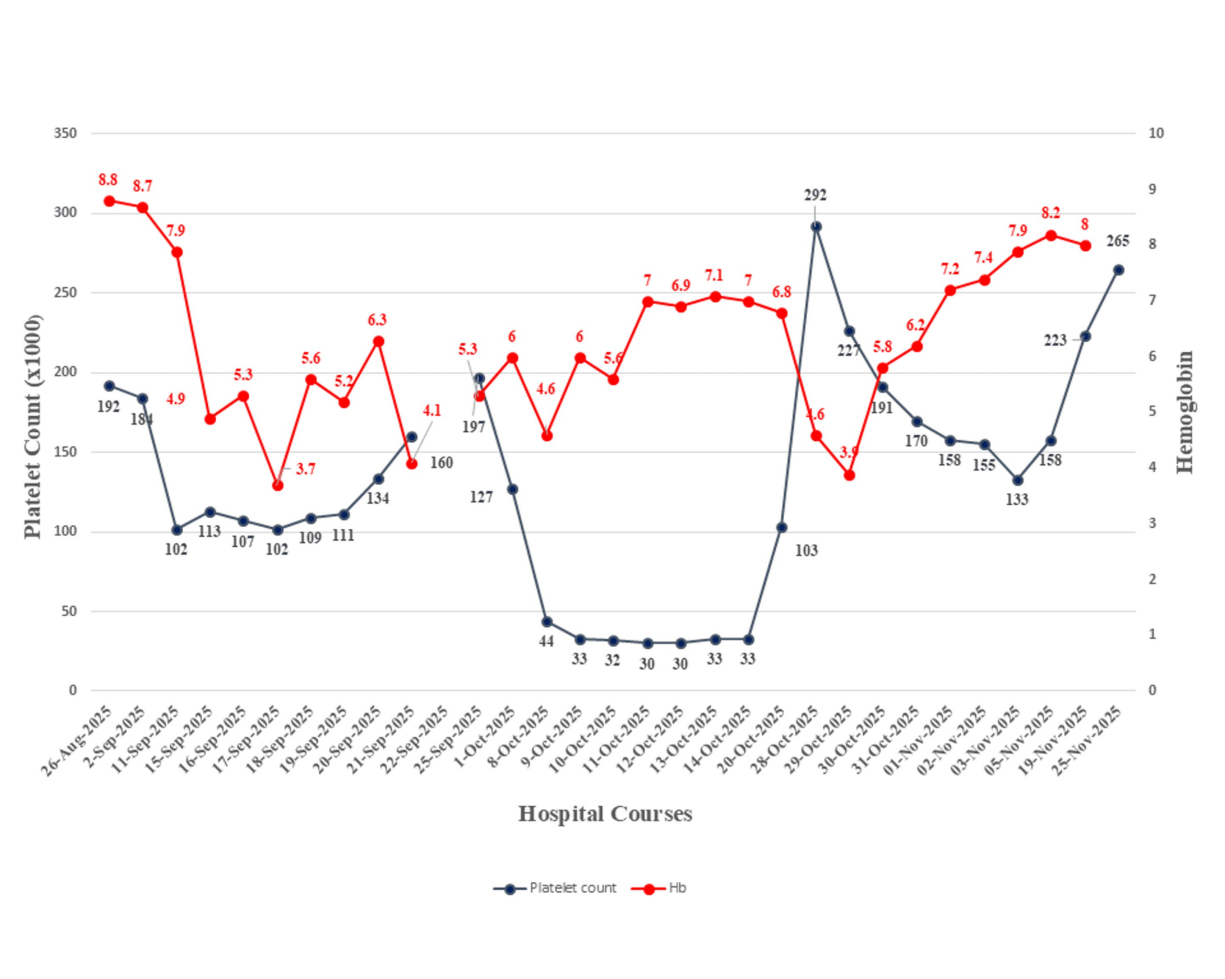
Clinical course of platelet count and hemoglobin during three hospitalizations.

During the first two hospitalizations, the patient also developed severe thrombocytopenia, with a platelet nadir of approximately 30 ×10⁹/L, whereas platelet levels normalized during the third admission, ranging between 200 and 300 ×10⁹/L (Figure 2).

## Discussion

AIHA is a rare disorder with an estimated incidence of 0.8 to 3 per 100,000 persons per year [5], characterized by immune-mediated destruction of red blood cells by the host immune system. The evaluation of suspected AIHA requires a conventional, stepwise approach to anemia, integrating laboratory findings to confirm hemolysis while systematically excluding alternative etiologies, including nutritional deficiencies, bleeding, and bone marrow failure, before establishing an immune-mediated mechanism [5]. Subsequent diagnostic testing is essential to confirm immune-mediated hemolysis and guide subtype-directed management.

The clinical presentation and management of AIHA are heterogeneous and influenced by multiple factors, including the AIHA subtype, severity of hemolysis, underlying diseases, presence of comorbid conditions, bone marrow compensatory capacity, and concomitant fibrosis or dyserythropoiesis.

**Figure 3:**
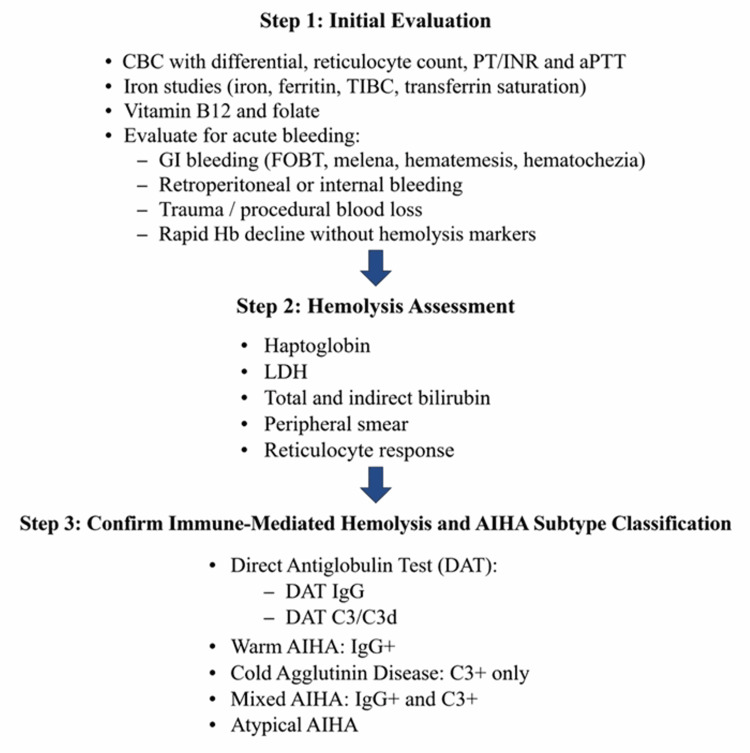
Stepwise diagnostic approach to anemia with evaluation for autoimmune hemolytic anemia. CBC, complete blood count; PT/INR, prothrombin time/international normalized ratio; aPTT, activated partial thromboplastin time; TIBC, total iron-binding capacity; FOBT, fecal occult blood test; GI, gastrointestinal; Hb, hemoglobin; LDH, lactate dehydrogenase; DAT, direct antiglobulin test; AIHA, autoimmune hemolytic anemia; IgG, immunoglobulin G; C3/C3d, complement component 3/complement component 3d.

In this case, the patient presented with acute severe hemolytic anemia in the setting of multiple comorbid conditions, including stage IV chronic kidney disease, HIV infection, and recent chemotherapy exposure. A structured, stepwise approach to anemia (Figure 3) demonstrated normal iron indices, vitamin B12, and folate levels, and gastrointestinal bleeding was considered unlikely given a negative fecal occult blood test. The rapid decline in hemoglobin from 8.7 g/dL to 4.7 g/dL over four days, together with low haptoglobin, markedly elevated LDH, and hyperbilirubinemia, strongly supported an active hemolytic process.

Further evaluation of hemolysis etiology using the direct antiglobulin test confirmed an immune-mediated mechanism. Immune-related hemolysis can be categorized as warm AIHA, cold agglutinin disease, or mixed AIHA based on DAT patterns of IgG and/or C3/C3d positivity, or alternatively classified as primary (idiopathic) or secondary to underlying conditions such as lymphoproliferative disorders, infections, drug exposure, or autoimmune diseases, including systemic lupus erythematosus [6]. Warm AIHA is typically characterized by DAT IgG positivity, occasionally with low-titer complement involvement, whereas cold forms demonstrate isolated complement (C3/C3d) positivity with high-titer cold agglutinins. Mixed AIHA is defined by concurrent IgG and complement positivity. Rarer forms include mixed AIHA with high thermal-amplitude cold agglutinins. Prior studies have also emphasized the role of cold agglutinin titers and autoagglutination at 20 °C in distinguishing mixed AIHA from warm AIHA [1]. However, cold agglutinin titers and thermal amplitude testing were not formally assessed in this case, which represents a limitation of our report.

Accurate identification of the AIHA subtype is critical, as it directly guides management strategies. Treatment options include corticosteroids, IVIG, and B-cell-directed therapies (rituximab alone or in combination with bendamustine or fludarabine), as well as splenectomy for refractory warm AIHA. In contrast, CAD management focuses on cold avoidance, complement inhibition (e.g., sutimlimab, an anti-C1s monoclonal antibody), and B-cell-directed therapy, as corticosteroids are generally ineffective and splenectomy is unlikely to be beneficial due to predominant hepatic clearance of C3b-opsonized erythrocytes. Supportive red blood cell transfusion remains an essential component of care depending on anemia severity and clinical stability [3].

In this patient, DAT positivity for both IgG and C3/C3d was consistent with mixed AIHA. Despite initial treatment with pulse-dose dexamethasone followed by IVIG (1 g/kg) and red blood cell transfusions, the patient experienced recurrent hemolytic episodes requiring multiple hospitalizations and ultimately required prolonged rituximab therapy over four weeks to achieve sustained hemoglobin stability. The etiology of secondary AIHA in this case was likely multifactorial, including HIV infection and exposure to immune checkpoint inhibition with retifanlimab. Although the patient’s HIV viral load was well controlled, HIV-associated immune dysregulation may still contribute to autoimmunity. While PD-1 inhibitors have been associated with warm AIHA, PD-L1 inhibitors have not consistently demonstrated an increased risk; nevertheless, AIHA occurring in patients receiving checkpoint inhibitors has been associated with increased morbidity and mortality [7]. The coexistence of immune thrombocytopenia consistent with Evans syndrome may further exacerbate disease severity in this setting, as Evans syndrome has been associated with higher relapse rates, greater treatment complexity, and worse clinical outcomes compared with isolated AIHA.

Several factors in this case are associated with an increased risk of AIHA relapse, including severe anemia at presentation (hemoglobin <6 g/dL), concurrent immune thrombocytopenia suggestive of Evans syndrome, complement involvement, and thermal characteristics of the autoantibody, which together have been shown to confer up to a four-fold increased risk of multiple relapses [4]. Furthermore, hemoglobin <6 g/dL at onset, Evans syndrome, requirement for multiple treatment lines, acute renal failure, and infectious complications have each been associated with a five- to eight-fold increase in mortality risk [4].

## Conclusions

AIHA is a rare but severe hematologic condition that requires a structured diagnostic evaluation to identify etiology, subtype, and appropriate treatment. Mixed AIHA, particularly in the setting of HIV, PD-1 inhibitor therapy, CKD, and metastatic cancer, poses diagnostic and therapeutic challenges and carries a high risk of relapse. This case underscores the importance of early recognition, aggressive initial therapy, and timely escalation to rituximab in steroid-refractory disease. Further research is needed to clarify the pathophysiology and optimal management strategies for mixed AIHA, especially when triggered by immune checkpoint inhibitors or occurring in immunocompromised patients.
